# Novel chemokine-like activities of histones in tumor metastasis

**DOI:** 10.18632/oncotarget.11226

**Published:** 2016-08-11

**Authors:** Ruochan Chen, Yangchun Xie, Xiao Zhong, Yongmin Fu, Yan Huang, Yixiang Zhen, Pinhua Pan, Haichao Wang, David L. Bartlett, Timothy R. Billiar, Michael T. Lotze, Herbert J. Zeh, Xue-Gong Fan, Daolin Tang, Rui Kang

**Affiliations:** ^1^ Department of Infectious Diseases and State Key Laboratory of Viral Hepatitis, Xiangya Hospital, Central South University, Changsha, Hunan 410008, China; ^2^ Department of Surgery, University of Pittsburgh, Pittsburgh, Pennsylvania 15213, USA; ^3^ Department of Pneumology, Xiangya Hospital, Central South University, Changsha, Hunan 410008, China; ^4^ Laboratory of Emergency Medicine, The Feinstein Institute for Medical Research, Manhasset, New York 11030, USA; ^5^ Center for DAMP Biology, The Third Affiliated Hospital of Guangzhou Medical University, Guangzhou, Guangdong, 510150, China

**Keywords:** histone, TLR4, NF-κB, metastasis, hepatocellular carcinoma

## Abstract

Histones are intracellular nucleosomal components and extracellular damage-associated molecular pattern molecules that modulate chromatin remodeling, as well as the immune response. However, their extracellular roles in cell migration and invasion remain undefined. Here, we demonstrate that histones are novel regulators of tumor metastasis with chemokine-like activities. Indeed, exogenous histones promote both hepatocellular carcinoma (HCC) cell migration and invasion through toll-like receptor (TLR)4, but not TLR2 or the receptor for advanced glycosylation end product. TLR4-mediated activation of nuclear factor-κB (NF-κB) by extracellular signal-regulated kinase (ERK) is required for histone-induced chemokine (e.g., C-C motif ligand 9/10) production. Pharmacological and genetic inhibition of TLR4-ERK-NF-κB signaling impairs histone-induced chemokine production and HCC cell migration. Additionally, TLR4 depletion (by using TLR4^−/−^ mice and TLR4-shRNA) or inhibition of histone release/activity (by administration of heparin and H3 neutralizing antibody) attenuates lung metastasis of HCC cells injected via the tail vein of mice. Thus, histones promote tumor metastasis of HCC cells through the TLR4-NF-κB pathway and represent novel targets for treating patients with HCC.

## INTRODUCTION

Hepatocellular carcinoma (HCC) is the fifth most common solid tumor and the third leading cause of cancer-related mortality worldwide, particularly in Asia [1]. Curative resection has prolonged the five-year survival rate of HCC patients, although recurrence and metastasis following curative resection are sadly common [2]. Although tumor progression with metastasis is the main cause of death in HCC patients, which underlying regulatory mechanisms promote this process are still not fully understood [3].

The tumor microenvironment is recognized as a key factor in multiple stages of HCC [4]; it is composed of a several cell types and factors including stellate cells, myeloid and lymphoid cells, cytokines, chemokines, and growth factors, as well as damage-associated molecular pattern molecules (DAMPs). DAMPs are endogenous molecules that can be passively released by dead, dying, and injured cells or actively secreted by immune cells in response to inflammatory stimuli or environmental stress [5, 6]. Although DAMPs are elevated in the serum of cancer patients, they may have different biological effects during tumorigenesis and interfere with the efficacy of cancer therapy via binding to respective receptors important for cancer progression [7, 8].

The release of nuclear DAMPs, including high mobility group box 1 (HMGB1), histones, and DNA, link genomic instability and inflammation [9, 10], two of the hallmarks of cancer [11]. Within the nucleus, nuclear DAMPs interact to regulate nucleosome stability. In the extracellular space, they can be recognized by pattern recognition receptors [e.g., toll-like receptors (TLRs) and the receptor for advanced glycosylation end product (RAGE)] involved in inflammation and immunity [12, 13]. Aberrant activation of cell death, as well as nuclear DAMP signaling, contributes to the pathogenesis of several liver diseases [14, 15].

Intracellular histones play key roles in the epigenetic modulation of tumorigenesis and drug resistance in multiple cancers, including HCC [16, 17]. However, the pathological role of extracellular histones in HCC remains unknown. In this study, we demonstrate that extracellular histones are a novel trigger of HCC cell migration and invasion. Furthermore, we found that TLR4 (but not TLR2 or RAGE)-mediated nuclear factor-κB (NF-κB) activation is required for the chemokine-like activity of histones in HCC cells. Pharmacological and genetic inhibition of the histone-TLR4-NF-κB pathway limits HCC cells metastasis *in vitro* and *in vivo*. These findings improve our understanding of the mechanisms of metastasis and open new opportunities for the treatment of HCC patients.

## RESULTS

### Circulating nucleosome levels are elevated in HCC patients

**Figure 1:**
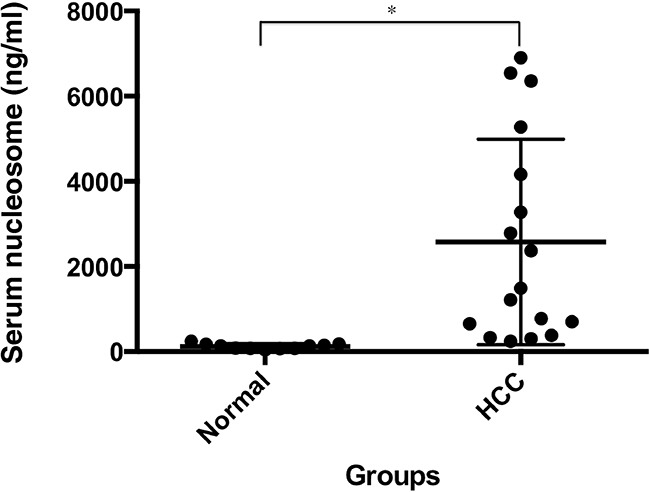
Serum nucleosomal levels are significantly increased in HCC patients Serum nucleosome levels were measured using ELISA in HCC patients (n=17) and healthy individuals (n=11). *p < 0.01.

To examine the role of extracellular histones in HCC, we first examined the level of circulating nucleosomes in serum samples from HCC patients (n=17, Supplementary Table S1) and a healthy control group (n=11) via ELISA analysis (Figure 1). The median plasma nucleosome concentration in HCC patients was 1492.32 ng/mL (min=240.40 ng/mL and max=6358.02 ng/mL), which was 10.66-fold higher than that of the control group (median=139.98 ng/mL; min=41.01 ng/mL; max=243.36 ng/mL; and p < 0.01). These findings indicate an important potential pathogenic role of circulating nucleosomes, including histone and DNA, in the development of HCC.

### Histones induce HCC cell migration and invasion

**Figure 2:**
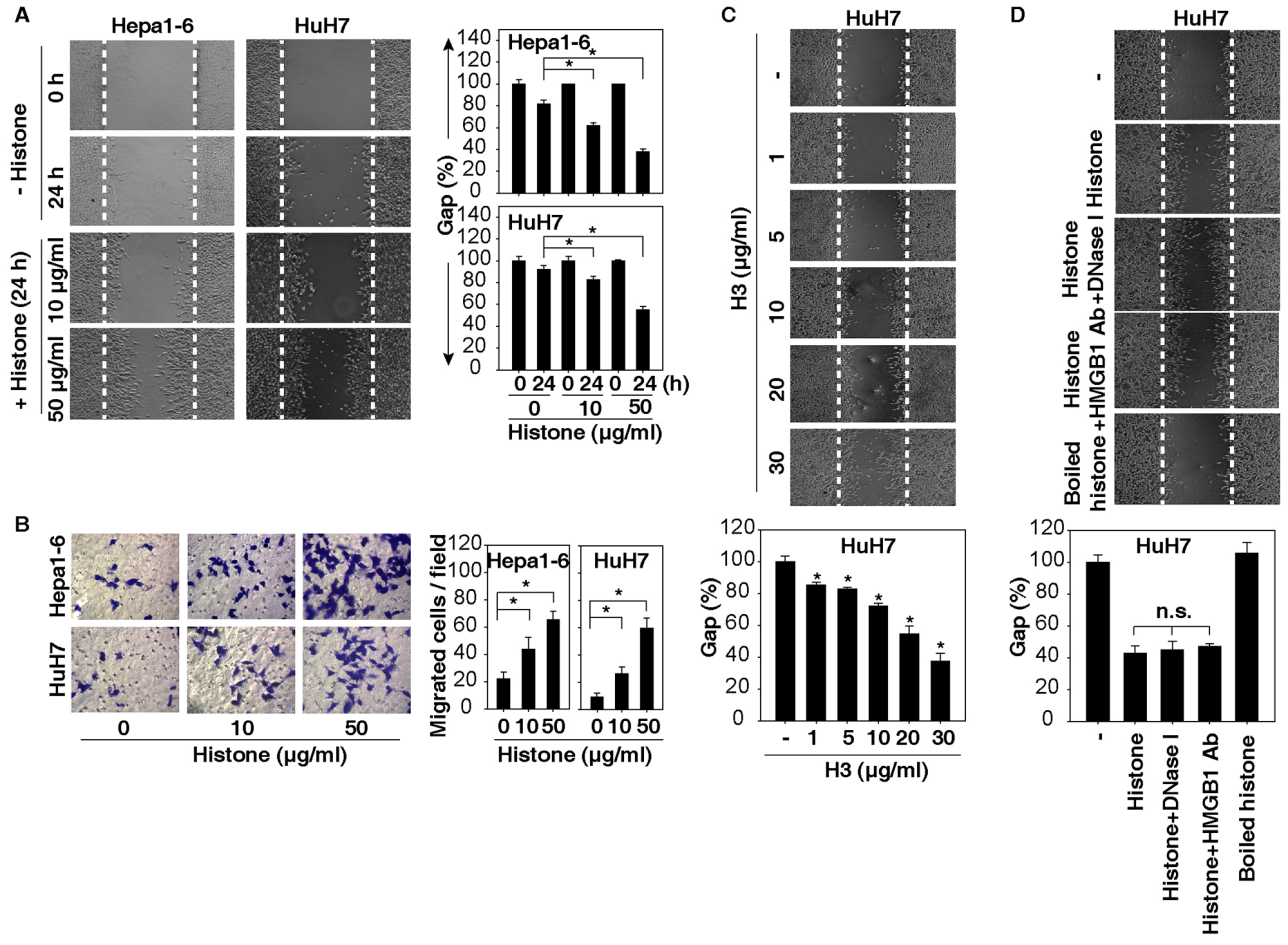
Histones induce HCC cell migration and invasion **A, B.** Histones dose-dependently promoted HCC cell migration and invasion at 24 hours as demonstrated by wound healing (A) and Transwell (B) assays (n=3, *, p<0.05). **C.** H3 dose-dependently promoted HuH7 cell migration at 24 hours as demonstrated by wound healing assays. **D.** HuH7 cells were treated with histone (50 μg/ml) in the absence or presence of DNase I (1 U/ml) or HMGB1-neutralizing antibody (20 μg/ml) for 24 hours, and cell migration was tested using wound healing assays (n=3, *, p<0.05 versus untreated group).

To determine whether histones induce HCC cell migration and invasion, we treated Huh7 (a human HCC cell line) and Hepa1-6 (a mouse HCC cell line) with highly purified histones including H1, H2A, H2B, H3, and H4 at 10-50 μg/ml as previously reported [18]. These histones had extremely low levels of endotoxins (<0.1 EU/ml by Limulus Amebocyte Lysate Testing). Significantly, histones dose-dependently promoted cell migration and invasion, as judged by wound healing (Figure 2A) and Transwell (Figure 2B) assays. Exogenous H3 alone dose-dependently induced HCC cell migration (Figure 2C). To determine whether histone-mediated cell migration was due to contaminating DNA or HMGB1, we treated Huh7 cells with histones in the presence of DNase and an HMGB1-neutralizing antibody, which did not alter histone-induced cell migration (Figure 2D). In contrast, boiled histones lost their chemokine-like activity in HCC cells (Figure 2D), confirming that histones have the ability to induce HCC cell migration and invasion, which are not dependent on the presence of DNA and HMGB1 as their predominant binding partners. Moreover, 0.1 EU/ml endotoxins did not induce Huh7 cell migration (data not shown), suggesting that histone-induced cell migration is not dependent on their extremely low levels of endotoxins present.

### Activation of NF-κB by ERK contributes to histone-induced HCC cell migration and invasion

**Figure 3:**
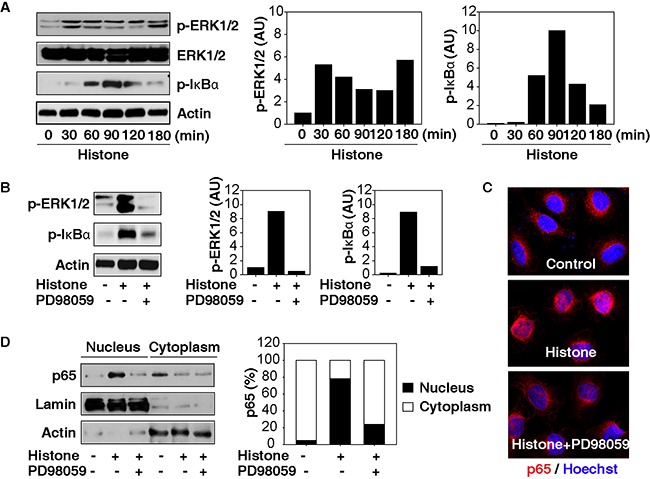
MAPK/ERK contributes to histone-induced NF-κB activation in HCC cells **A.** Histones (50 μg/ml) induce phosphorylation of ERK and IκB in Hepa1-6 cells. **B-D.** The MEK inhibitor PD98059 (20 μM) inhibited the phosphorylation of ERK and IκB (B) and the nuclear translocation of p65 in Hepa1-6 cells following histone treatment (50 μg/ml, 60 min).

Given that an abnormal extracellular signal-regulated kinase (ERK) and NF-κB pathway has been implicated in tumorigenesis and therapy in HCC [19, 20], we assayed the activation of the ERK-NF-κB pathway in HCC cells following histone treatment. Histones strongly induced phosphorylation of ERK and IκB (Figure 3A) and were prevented by PD98059, an inhibitor of ERK's upstream kinase (namely MAP kinase, termed MEK) (Figure 3B). As expected, histone-induced translocation of NF-κB p65 from the cytosol to the nucleus was also limited by PD98059 (Figure 3C and 3D). These findings suggest that MAPK/ERK facilitates NF-κB activation in HCC cells following histone treatment.

**Figure 4:**
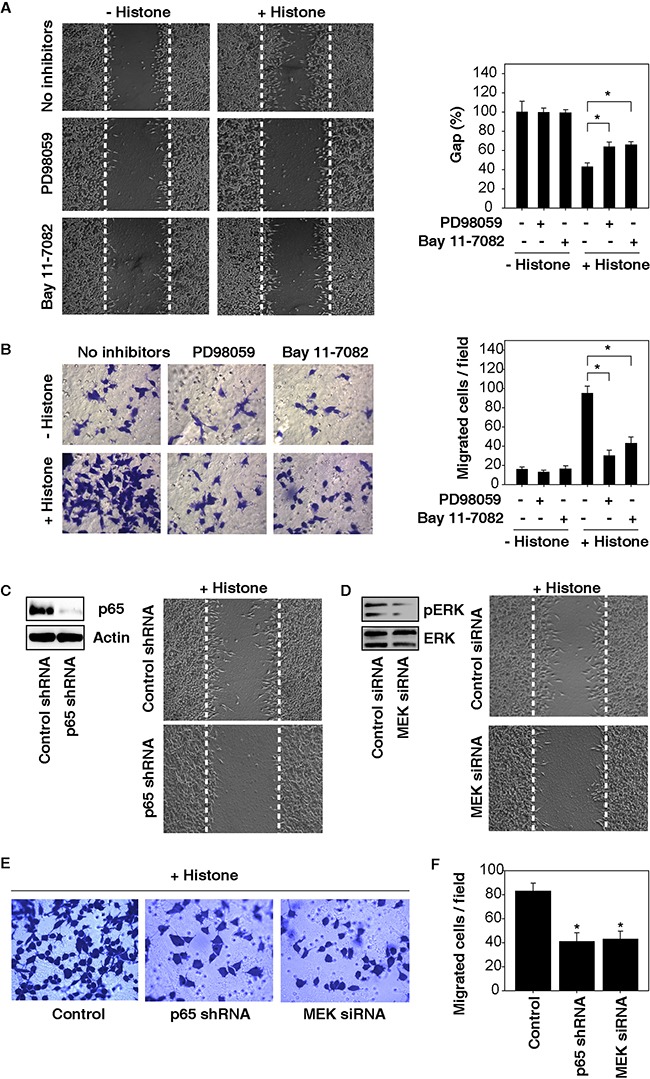
Activation of the MAPK/ERK-NF-κB pathway contributes to histone-induced HCC cell migration and invasion **A.** Both MEK (PD98059, 20 μM) and NF-κB (Bay11-7082, 10 μM) inhibitors suppressed histone (50 μg/ml, 24 hours)-induced Hepa1-6 cell migration **A.** and invasion **B.** (n=3, *, p<0.05). **C-F.** Knockdown of p65 and MEK by RNAi inhibited histone (50 μg/ml, 24 hours)-induced HCC cell migration (C, D) and invasion (E, F) (n=3, *, p<0.05, versus control group).

Next, we investigated whether blocking the ERK-NF-κB pathway affects histone-induced cell migration and invasion. Both MEK (PD98059) and NF-κB (Bay11-7082) inhibitors suppressed histone-induced cell migration and invasion in HCC cells (Figure 4A and 4B). Moreover, knockdown of p65 (a core component of the NF-κB pathway) and MEK by RNAi also significantly inhibited histone-induced HCC cell migration and invasion (Figure 4C-4F). These findings indicate that activation of the NF-κB pathway by MAPK/ERK is responsible for histone-induced HCC cell migration and invasion.

### TLR4 is required for histone-induced NF-κB activation and cell migration in HCC cells

**Figure 5:**
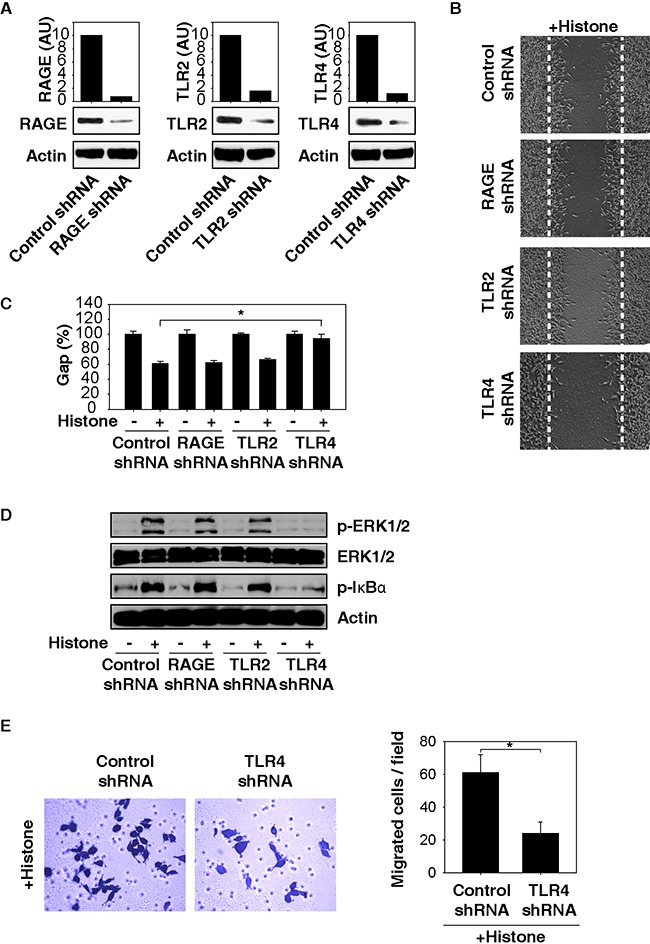
TLR4 is required for histone-induced NF-κB activation and HCC cell migration Knockdown of TLR4 (but not TLR2 and RAGE) by RNAi **A.** inhibited histone (50 μg/ml)-induced Hepa1-6 cell migration **B, C.** phosphorylation of ERK and IκB **D.** and cell invasion **E.** (n=3, *, p<0.05).

Several receptors, including TLRs and RAGE, mediate biological activities of extracellular histones in individual cell types [21–23]. To better understand the mechanisms of histone-induced cell migration, we suppressed the expression of TLR2, TLR4, or RAGE by RNAi in HCC cells. Knockdown of TLR4 (but not TLR2 and RAGE) with specific shRNA (Figure 5A) significantly inhibited histone-induced cell migration (Figure 5B and 5C) and decreased phosphorylation of ERK and IκB (Figure 5D). Consistently, knockdown of TLR4 by RNAi inhibited histone-induced cell invasion (Figure 5E). Thus, TLR4 seemed to play a major role in mediating histone-induced ERK-NF-κB signaling activation and subsequent cell migration in HCC cells.

### Histones promote chemokine production and release

**Figure 6:**
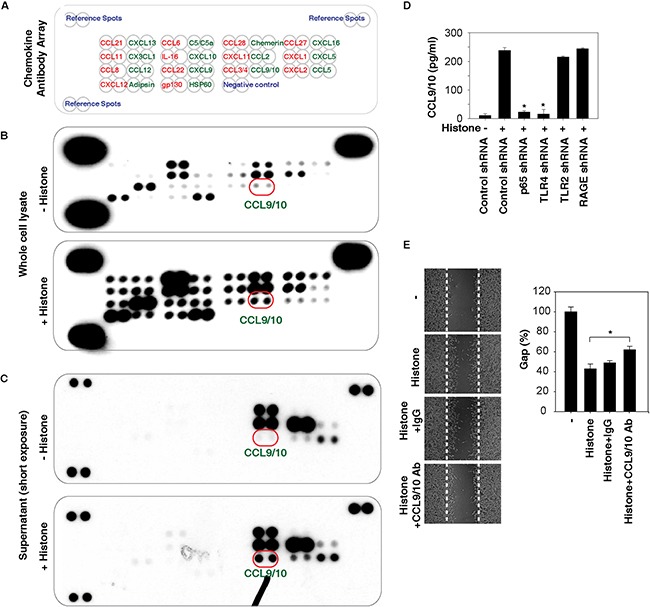
Histones promote chemokine production and release **A-C.** Histones (50 μg/ml, 24 hours) induced chemokine production and release as demonstrated with a Proteome Profiler™ Antibody Array in Hepa1-6 cells. **D.** Knockdown of NF-κB p65 and TLR4 (but not TLR2 and RAGE) in Hepa1-6 cells inhibited histone (50 μg/ml, 24 hours)-induced CCL9/10 release as demonstrated by ELISA assay (n=3, *, p<0.05 versus control shRNA group). **E.** Anti-CCL9/10 neutralizing antibody (1 mg/ml) partly inhibited histone (50 μg/ml, 24 hours)-induced Hepa1-6 cell migration (n=3, *, p<0.05).

The activation of NF-κB occupies an important role in the transcription of genes that encode chemokines [24]. Given that histones are potent inducers of NF-κB activity, we next addressed whether histone affects chemokine expression and release using a Proteome Profiler™ Antibody Array in HCC cells. This Proteome Profiler Chemokine Array Kit is a membrane-based sandwich immunoassay to simultaneously test 31 individual chemokines (Figure 6A). Compared with the untreated group, histone stimulated the production (Figure 6B) of multiple chemokines. Among them, the release of C-C motif ligand 9/10 (CCL9/10) was significantly increased following histone treatment (Figure 6C). Consistently, the histone-induced CCL9/10 release was confirmed by quantitative ELISA assays and found to be blocked by knockdown of NF-κB p65 and TLR4 (but not TLR2 and RAGE) (Figure 6D). Moreover, anti-CCL9/10 neutralizing antibody also partly inhibited histone-induced HCC cell migration (Figure 6E), suggesting that the release of chemokines such as CCL9/10 contributes to histone-induced cell migration.

### Histone-TLR4 pathway mediates lung metastasis of HCC cells in vivo

**Figure 7:**
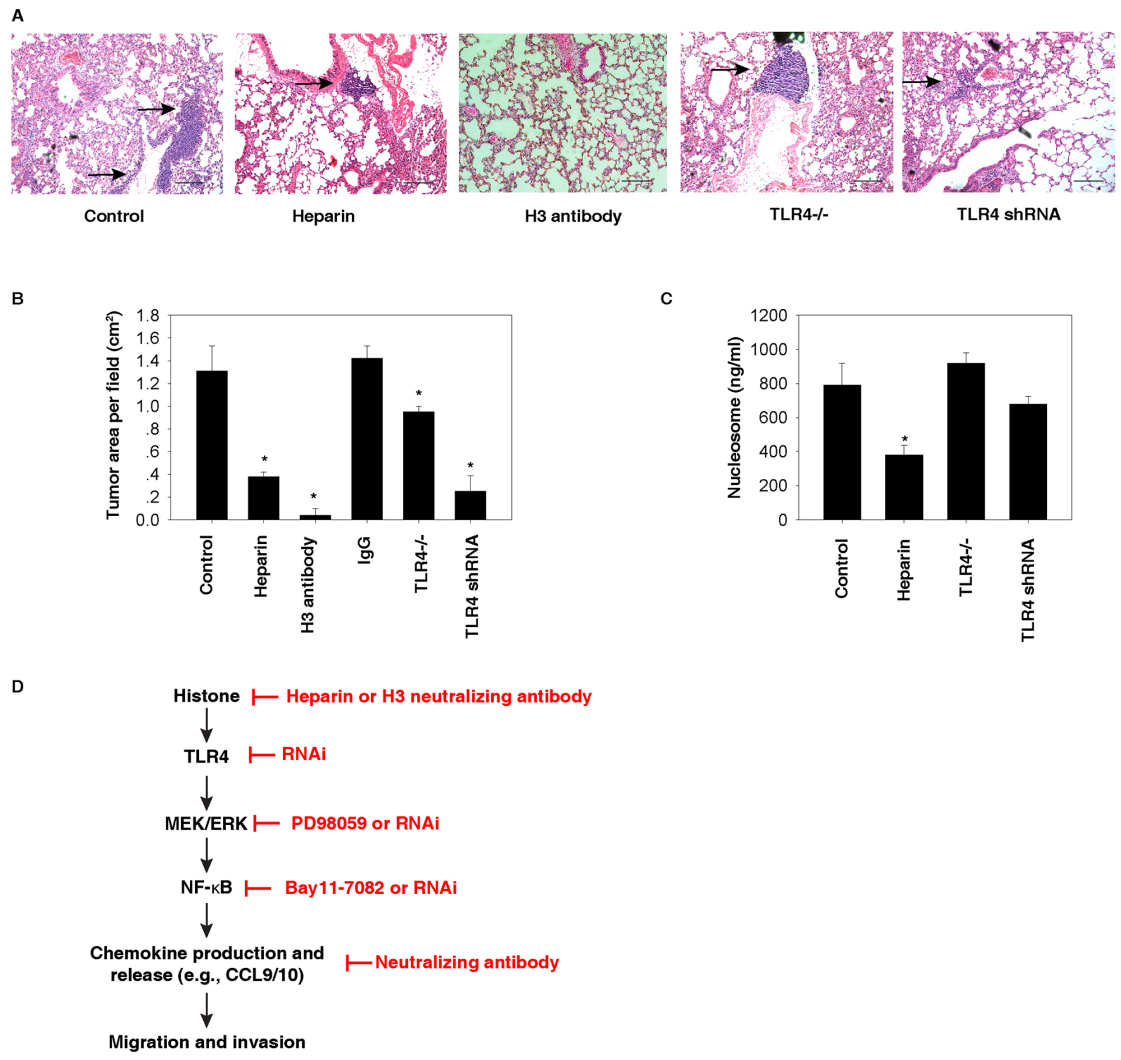
Histone-TLR4 pathway mediates lung metastasis of HCC cells *in vivo* **A-B.** Compared with the control group, TLR4 depletion (by using TLR4^−/−^ mice or TLR4 knockdown cells) or inhibition of histone release (by administration of 10 mg/kg heparin or 10 mg/kg H3 neutralizing antibody) limited the formation of lung metastasis (as shown in arrow) in mice based on tail vein injection of 3×10^6^ Hepa1-6 cells (N=5 mice/group, *, p<0.05 versus control group). In contrast, control IgG (10 mg/kg) did not inhibit the formation of lung metastasis (B). **C.** Serum nucleosome levels were reduced after treatment with heparin in wildtype, but not in TLR4^−/−^ mice (N=5 mice/group, *, p<0.05 versus control group). **D.** Conceptual relationships between histone and tumor metastasis. Histone is a nuclear DAMP and can be released during cell injury or death. Once released, histone can promote cell migration and invasion through the TLR4-ERK-NF-κB pathway, which induces chemokine production and release.

To further determine the role of histones and TLR4 in tumor metastasis *in vivo*, we utilized a murine model for pulmonary metastasis by intravenous injection of mouse Hepa1-6 cells. Compared with the control group, TLR4 depletion (by using TLR4^−/−^ mice or TLR4 knockdown cells) or inhibition of histone release [by administration of heparin [25, 26]] or activity [by administration of H3 neutralizing antibody [9]] limited the formation of lung metastasis in mice (Figure 7A and 7B). Compared with TLR4^−/−^ mice, knockdown of TLR4 in Hepa1-6 cells resulted in more inhibition of lung metastasis in mice (Figure 7B), suggesting that TLR4 expression in HCC cells is more important than other cells in the host to mediate tumor metastasis. Administration of control IgG (10 mg/kg) did not inhibit the formation of lung metastasis of HCC cells (Figure 7B). As expected, serum nucleosome levels were reduced after treatment with heparin, but not in TLR4^−/−^ mice (Figure 7C). Collectively, these findings suggest that blocking the histone-TLR4 pathway inhibited lung metastasis of HCC cells *in vivo*.

## DISCUSSION

HCC is an aggressive tumor with several different treatment options, depending on the stage at presentation. Despite advances in the treatment of patients with HCC, the prognosis of those with extrahepatic metastasis remains extremely poor. In the present study, we demonstrated that histones, as nuclear DAMPs, promote the migration and invasion of HCC cells via activation of NF-κB-dependent chemokine production and release (Figure 7D). This extracellular activity of histones in HCC cells requires the involvement of TLR4, but not other DAMP receptors.

Genomic instability, a characteristic of most cancers, leads to an increase in genetic alterations and heightened programmed and unscheduled cell death, enabling the acquisition of additional hallmarks of cancer [27]. Histones are highly conserved nucleosomal structure proteins and play fundamental roles in chromatin remodeling and gene regulation. Alterations in epigenetic regulations by histone modification contribute to the development of many cancers, including HCC [16, 17]. In addition to their nuclear function, extracellular histones in conjunction with DNA serve as DAMPs, contributing to the pathogenesis of inflammatory diseases and cancer [28]. In patients with several types of advanced cancer (e.g., breast, cervical, lung, pancreatic, and colorectal cancer), serum nucleosomal levels are significantly higher when compared with healthy individuals and are directly related to therapeutic response [29–32]. Similarly, we found that serum nucleosome levels are also elevated in HCC patients compared with normal healthy controls. Further clinical studies are required to determine whether circulating nucleosomes could be a useful biomarker for early diagnosis, therapy response monitoring, and assessment of prognosis of HCC in patients.

Deregulated or abnormal cell death is associated with DAMP release, defining the pro-inflammatory properties of cell death [6, 12]. In addition to mediating the altered inflammatory and metabolic response, several nuclear DAMPs, including HMGB1 [33] and histones (current study), can also promote tumor cell migration, invasion, and metastasis. The extracellular activities of these nuclear DAMPs are regulated by their binding-receptors (e.g., RAGE and TLRs) [34, 35]. For example, we previously demonstrated that HMGB1 promotes the migration and invasion of HCC cells in a RAGE-dependent manner [36]. Furthermore, RAGE is similarly required for HMGB1-induced alterations in bioenergetics and nuclear DNA-HMGB1-histone complex-induced cell death in pancreatic cancer cells [37] and macrophages [38]. In contrast, here we demonstrate that only TLR4 (but not RAGE and TLR2) is required for histone-induced HCC cell migration, invasion, and metastasis *in vitro* and *in vivo*. Another study indicates that TLR9 contributes to histone-mediated hepatic ischemia/reperfusion injury [23]. Thus, multiple receptors can mediate histone responses under different conditions. In addition to DAMPs, TLR4 also binds to pathogen-associated molecular patterns (PAMPs), particularly the lipopolysaccharide (LPS) of Gram-negative bacteria, to trigger an inflammatory response. Collectively, TLR4 expressed in tumor or non-tumor cells within the tumor microenvironment uniformly contributes to HCC development in response to microbial infection [39, 40]. HCC patients whose tumors express high levels of DAMP receptors, including TLR4, have a poor prognosis [41]. Moreover, mutations or polymorphisms of the TLR4 gene are closely associated with liver carcinogenesis or malignancy [42]. Thus, TLR4 signaling appears to be important for hepatocarcinogenesis by recognizing DAMPs (e.g., histone) and PAMPs (e.g., LPS).

Our study also indicates that TLR4-mediated NF-κB activation is required for chemokine production in the histone-mediated invasion and metastasis of HCC. Chemokines are a group of small, structurally-related proteins, usually acting as chemoattractants to guide HCC cell migration, invasion, and metastasis [43, 44]. NF-κB controls various inflammation and expression of survival-associated genes in HCC tumorigenesis. NF-κB is also a master transcriptional regulator of gene expression of tumor-associated chemokines in promoting tumor growth and angiogenesis [45]. Histones significantly induced the production and release of a number of chemokines (e.g., CCL9/10). Knockdown of NF-κB p65 and TLR4 diminished histone-induced CCL9/10 release, suggesting that the TLR4-NF-κB pathway is required for histone-induced chemokine production. CCL9/10 has also been implicated in chronic hepatitis-associated hepatic fibrosis [46]. We found that inhibition of CCL9/10 activity by neutralizing antibody partly inhibited histone-induced migration and invasion, suggesting a potential role of CCL9/10 in HCC.

In summary, our work indicates a critical role of extracellular histones as nuclear DAMPs in mediating HCC cell migration and invasion via activation of the TLR4-NF-κB pathway *in vitro*. The process of lung metastasis by HCC cells is also drastically abolished by blocking histone activity and release *in vivo*. These findings provide novel insight into the function of nuclear DAMPs in the tumor microenvironment and shed light on the development of a novel histone-targeting strategy in the treatment of patients with HCC [47].

## MATERIALS AND METHODS

### Antibodies and reagents

The antibodies to p-IκBα (#2859), p-ERK1/2 (#4370), ERK (#9102), p65 (#4764), and actin (#3700) were obtained from Cell Signaling Technology (Danvers, MA, USA). The antibodies to Lamin B1 (#ab16048), TLR4 (#ab13867), RAGE (#Ab37647), and HMGB1 (#ab18256) were obtained from Abcam (Cambridge, MA, USA). The antibody to TLR2 (#SC-16237) was obtained from Santa Cruz Biotechnology (Dallas, Texas, USA). The antibody to CCL9/10 (#MAB463) was obtained from R&D Systems Inc. (Minneapolis, MN, USA). High purity histone proteins (#10223565001), including H3 (#11034758001), were obtained from Roche Life Science (Stockholm, Sweden). H3 neutralizing antibody (#ab1791) and control IgG were obtained from Abcam as we previously described [9]. Other inhibitors and reagents were obtained from Sigma (St. Louis, MO, USA).

### Patient serum samples

Serum samples fromHCC patients and healthy controls were collected from Xiangya Hospital, Central South University. Collection of the samples was approved by Xiangya Hospital's Institutional Review Board. Serum nucleosomes were quantified using the ELISA kit (#11774425001) from Roche Diagnostics (Germany).

### Cell culture

Hepa1-6 and HuH7 cells were grown in Dulbecco's Modified Eagle's Medium (DMEM) with 10% fetal bovine serum (FBS), 2 mM L-glutamine, and 100 U/ml of penicillin and streptomycin.

### Western blot analysis

Western blot was used to analyze protein expression as described previously [48, 49]. In brief, after extraction, proteins in cell lysates were first resolved by SDS-polyacrylamide gel electrophoresis and then transferred to nitrocellulose membrane and subsequently incubated with the primary antibody. After incubation with peroxidase-conjugated secondary antibodies, the signals were visualized by enhanced chemiluminescence (Pierce, Rockford, IL, USA) according to the manufacturer's instructions.

### RNAi

Specific p65-shRNA, RAGE-shRNA, TLR2-shRNA, TLR4-shRNA, and control-shRNA were purchased from Sigma-Aldrich. MEK-siRNA and control-siRNA were purchased from Santa Cruz Biotechnology. Cells were seeded in six-well plates at a density of 5×10^5^ cells/well to achieve a confluence of 70% overnight. The transfection was done using FuGENE 6 (Roche Life Science) according to the manufacturer's instructions. The transfection efficiency by RNAi was confirmed by western blot.

### Wound healing assay

Wound healing assay was performed to evaluate HuH7 and Hepa1-6 cell motility in response to extracellular histones. A straight line was drawn across the bottom of 12-well plates. HCC cells were trypsinized and resuspended in non-serum containing DMEM. A total of 5×10^5^ cells were plated into the plate overnight to achieve subconfluence. After scraping cells with a sharp tip vertically to the marked lines and washing with phosphate buffered saline (PBS) three times to get rid of floating cells, a cell-free space was created. The width of the cell-free space was measured after 24 h.

### Transwell migration and invasion assay

Cell migration was also studied using Corning Transwell polycarbonate membrane inserts with 24 pores (pore size 8.0 μm, membrane diameter 6.5 mm). For cell invasion assay, we used the same protocol, except that the Transwell inserts were coated with a layer of basement membrane extract to mimic extracellular matrix. HCC cells in the logarithmic growth phase were starved in serum-free media 24 hours prior to assay. After incubating cells for 24 hours in serum-free media, cells were harvested and counted. The cells were washed with sterile PBS and suspended at 1×10^6^/ml in serum-free medium. 100 μl cells were added into the upper chamber and 650 μl DMEM medium with 10% FBS and other reagents was used as a chemoattractant in the lower chamber. Cell migration and invasion was allowed to progress for 24h at 37°C with 5% CO_2_. Afterward, the matrix gel and cells on the top membrane surface were removed with a cotton swab. Transwell membranes were then stained with 0.1% crystal violet and light microscopy was used to photograph the cells that migrated through the membrane. The number of migrating cells per field was counted under microscopy.

### Chemokine assay

The production or release of chemokines was assayed using a Proteome Profiler™ Antibody Chemokine Array Kit (#ARY020) from R&D Systems Inc. according to the manufacturer's instructions. The release of CCL9/10 was assayed using ELISA (#EMCCL9) from Thermo Fisher Scientific Inc. according to the manufacturer's instructions.

### Experimental animal metastasis model

All animal experiments were approved by the Institutional Animal Care and Use Committees from Xiangya Hospital and the University of Pittsburgh and performed in accordance with Association for Assessment and Accreditation of Laboratory Animal Care guidelines (http://www.aaalac.org).

To determine the role of TLR4 in metastasis, stable TLR4 knockdown or wildtype Hepa1-6 cells (3×10^6^ in 0.3 mL PBS) were injected into the tail veins of TLR4^+/+^ or TLR4^−/−^ C57BL/6 mice (The Jackson Laboratory, USA). On day 56 following injection, lungs were removed and prepared for histopathology.

To determine the role of histones in metastasis, C57BL/6 mice were injected with Hepa1-6 cells (3 × 10^6^ cells/mouse) and treated with the histone inhibitor heparin (10 mg/kg intraperitoneally once every other day) [26] or H3 neutralizing antibody (10 mg/kg intraperitoneally once every other day) [9] or control IgG at day one for two weeks. On day 56 after the start of treatment, lungs were removed and assayed using histopathology.

### Statistical analysis

Unless otherwise indicated, data are expressed as means ± SD of three independent experiments. Unpaired Student's t tests were used to compare the means of two groups. One-way ANOVA was used for comparison among the different groups. When ANOVA was significant, *post hoc* testing of differences between groups was performed using the LSD test. A *p*-value < 0.05 was considered significant.

## SUPPLEMENTARY TABLE


