# Supplementary material for: Novel chemokine-like activities of histones in tumor metastasis
Source: Oncotarget. 2016 Aug 11;7(38):61728–40. doi: 10.18632/oncotarget.11226 (PMC5308686; doi:10.18632/oncotarget.11226)
Supplement: Supplementary file 1 [file oncotarget-07-61728-s001.pdf]

Novel chemokine-like activities of histones in tumor metastasis

SUPPLEMENTARY TABLE

Supplementary Table S1: Clinicopathological information of HCC patients

Clinicopathological features	Number
**Sex**	17
**Male**	13 (76.47%)
**Age (mean)**	51.59±9.42
**Cirrhosis**	8 (47.06%)
**Hepatitis (antibody)**	
HBV	10 (58.80%)
HCV	1 (5.88%)
**No. viral hepatitis**	
ETOH	0
NASH	1 (5.88%)
other	5 (29.41%)
**Tumor size**	
≥5 cm	11 (64.7%)
**No. of nodules**	
≥2	4(23.5%)
=1	13(74.5%)
**Child-Pugh score**	
>5	(29.41%)
**No. of lymph node**	
≥2	2(11.67%)
**Cellular differentiation**	
Well	0
Moderate	7 (41.18%)
Poor	1 (6%)
Without pathology	9 (52.94%)
**Venous invasion**	
present	6 (35.29%)
**Tumor stage**	
Stage 0	0
Stage I	3 (17.65%)
Stage II	4 (23.53%)
Stage III	1 (5.88%)
Stage IV	7 (41.18%)
